# Case Report: Epidermodysplasia verruciformis with multiple squamous and basal cell carcinomas

**DOI:** 10.3389/fmed.2025.1565977

**Published:** 2025-06-04

**Authors:** Zijing Zhang, Chuanbo Liu, Jinsheng Li

**Affiliations:** 1 The Fourth School of Clinical Medicine, Zhejiang Chinese Medical University, Hangzhou First People's Hospital, Hangzhou, China; 2 Department of Plastic and Cosmetic Surgery, Affiliated Hangzhou First People's Hospital, School of Medicine, Westlake University, Hangzhou, Zhejiang, China

**Keywords:** epidermodysplasia verruciformis, squamous cell carcinoma, basal cell carcinoma, histopathological examination, malignancy

## Abstract

Epidermodysplasia verruciformis (EV) is a rare genodermatosis characterized by chronic infection with human papillomavirus (HPV), leading to disseminated flat-topped papules and pityriasis versicolor-like lesions. Patients with EV are predisposed to developing non-melanoma skin cancers, particularly squamous cell carcinoma (SCC) in sun-exposed areas. Currently, there is no definitive cure for EV, and management remains largely supportive and symptomatic. We report a case of EV in a 38-year-old male, presenting with classical clinical features and histopathological findings, complicated by the simultaneous development of squamous cell carcinoma(SCC) and basal cell carcinoma (BCC). This case highlights the oncogenic potential of EV-associated HPV subtypes and the importance of regular dermatologic surveillance in affected individuals.

## 1 Introduction

Epidermodysplasia verruciformis (EV) is a rare genodermatosis caused by an increased susceptibility to certain subtypes of human papillomavirus (HPV) (1), typically manifesting as persistent common warts, flat warts, or pityriasis versicolor-like lesions. Affected individuals commonly present with multiple, small, slightly elevated papules, predominantly localized on the hands, feet, face, and neck. These lesions carry a significant risk of malignant transformation into squamous cell carcinoma (SCC), particularly in sun-exposed regions (2).

Epidermodysplasia verruciformis (EV) is typically inherited in an autosomal recessive manner, with cases linked to mutations in the EVER1/TMC6 and EVER2/TMC8 genes (3). Affected individuals may present with clinical manifestations at an early age, and skin malignancies develop in approximately 30% to 50% of EV patients, particularly squamous cell carcinoma (SCC) (4). Current therapeutic options for EV remain suboptimal, with common interventions including topical treatments, photodynamic therapy (PDT), and surgical excision. However, these approaches often fail to fully eradicate the cutaneous lesions and are associated with a high recurrence rate (5).

We present the clinical case of a 38-year-old male with a longstanding history of epidermodysplasia verruciformis (EV), which has progressed to multiple cutaneous malignancies, including squamous cell carcinoma (SCC) and basal cell carcinoma (BCC).

## 2 Case presentation

**Figure 1:**
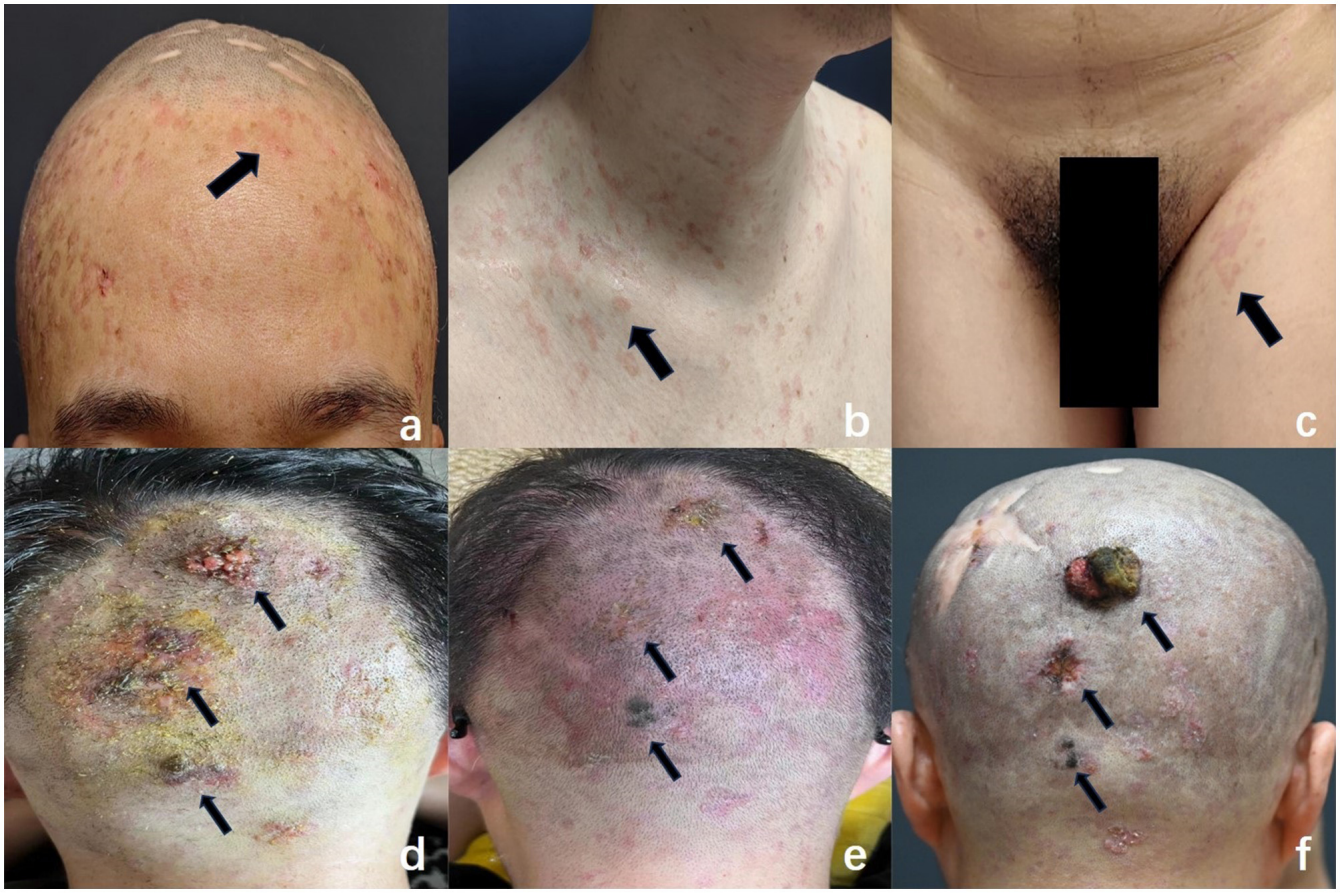
Characteristic clinical features of EV: flat, erythematous macules were observed on the forehead **(a)**, clavicular region **(b)**, and inguinal area **(c)** (arrows), resembling pityriasis versicolor. These lesions varied in size and exhibited irregular shapes, well-demarcated borders, and slight central depression. **(d)** Multiple initial cauliflower-like lesions were noted in the occipital region (arrows). **(e)** Following photodynamic therapy, the lesions regressed significantly and showed near-complete resolution (arrows). **(f)** Recurrence of lesions: new elevated masses re-emerged in the previously treated area, varying in size, with the largest measuring 3 cm × 2.5 cm × 1 cm. These lesions exhibited a cauliflower-like appearance, indistinct margins, surface ulceration and erosion, and a tendency to bleed upon contact.

A 38-year-old male with a 30-year history of epidermodysplasia verruciformis (EV) presents with distinct clinical manifestations, including erythematous, irregularly shaped, flat papules located on the scalp, clavicles, hands, and inguinal region (Figures 1a–c). The patient has a prior history of squamous cell carcinoma (SCC) of the scalp, for which he underwent surgical excision approximately 10 years ago, with subsequent uneventful recovery and no further interventions required. Over the past two years, the patient has developed three progressively enlarging exophytic lesions on the occipital scalp, accompanied by erosion and bloody discharge (Figure 1d). Histopathological analysis of the biopsy specimen confirmed a diagnosis of squamous cell carcinoma.The patient had been undergoing regular photodynamic therapy (PDT) at another institution, which resulted in significant improvement of his condition, with gradual regression and healing of the cutaneous excrescences (Figure 1e). However, one year ago, the patient discontinued the photodynamic treatment, leading to the recurrence of the lesions at the original site. These lesions re-enlarged to the size of a quail egg, accompanied by erosion and ulceration (Figure 1f). The patient denies any other significant medical conditions or a family history of similar lesions, and consanguinity in his family is also denied. He reports no history of prolonged outdoor exposure or regular sun exposure.

The patient was in generally good health, with no significant abnormalities identified upon systemic examination. Dermatological assessment revealed the presence of diffuse or scattered, irregularly shaped, pale erythematous maculopapular lesions of varying sizes, located on the scalp, preclavicular region, groin, and the dorsal aspects of both hands. The lesions exhibited well-demarcated borders with central slight depressions (Figures 1a–c).

**Figure 2:**
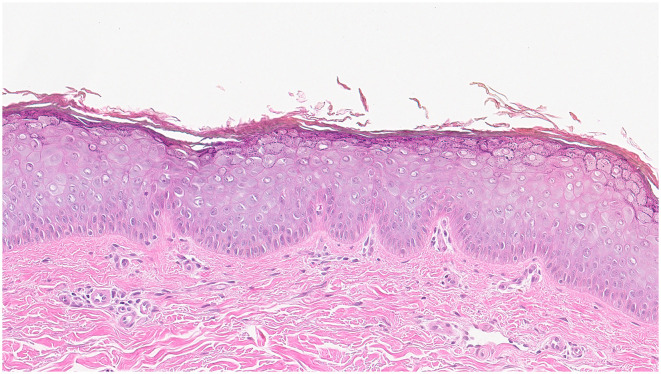
Histopathology of EV lesions: The epidermis demonstrated hyperkeratosis, thickening of the granular layer, and acanthosis. Numerous large vacuolated cells were observed in the upper two-thirds of the epidermis, while the lower spinous layer showed marked irregular thickening with keratinocytes displaying prominent gray-blue cytoplasmic changes. A mild perivascular lymphocytic infiltrate was noted in the superficial dermis, supporting the diagnosis of EV.

A skin biopsy was performed on a lesion located on the patient's left forearm. Histopathological analysis revealed features consistent with Epidermodysplasia verruciformis (Figure 2). Microscopically, the epidermis demonstrated hyperkeratosis, thickening of the granular layer, and acanthosis. Numerous large vacuolated cells were observed in the upper two-thirds of the epidermis, while the lower spinous layer showed marked irregular thickening with keratinocytes displaying prominent gray-blue cytoplasmic changes. A mild perivascular lymphocytic infiltrate was noted in the superficial dermis, supporting the diagnosis of Epidermodysplasia verruciformis.

**Figure 3:**
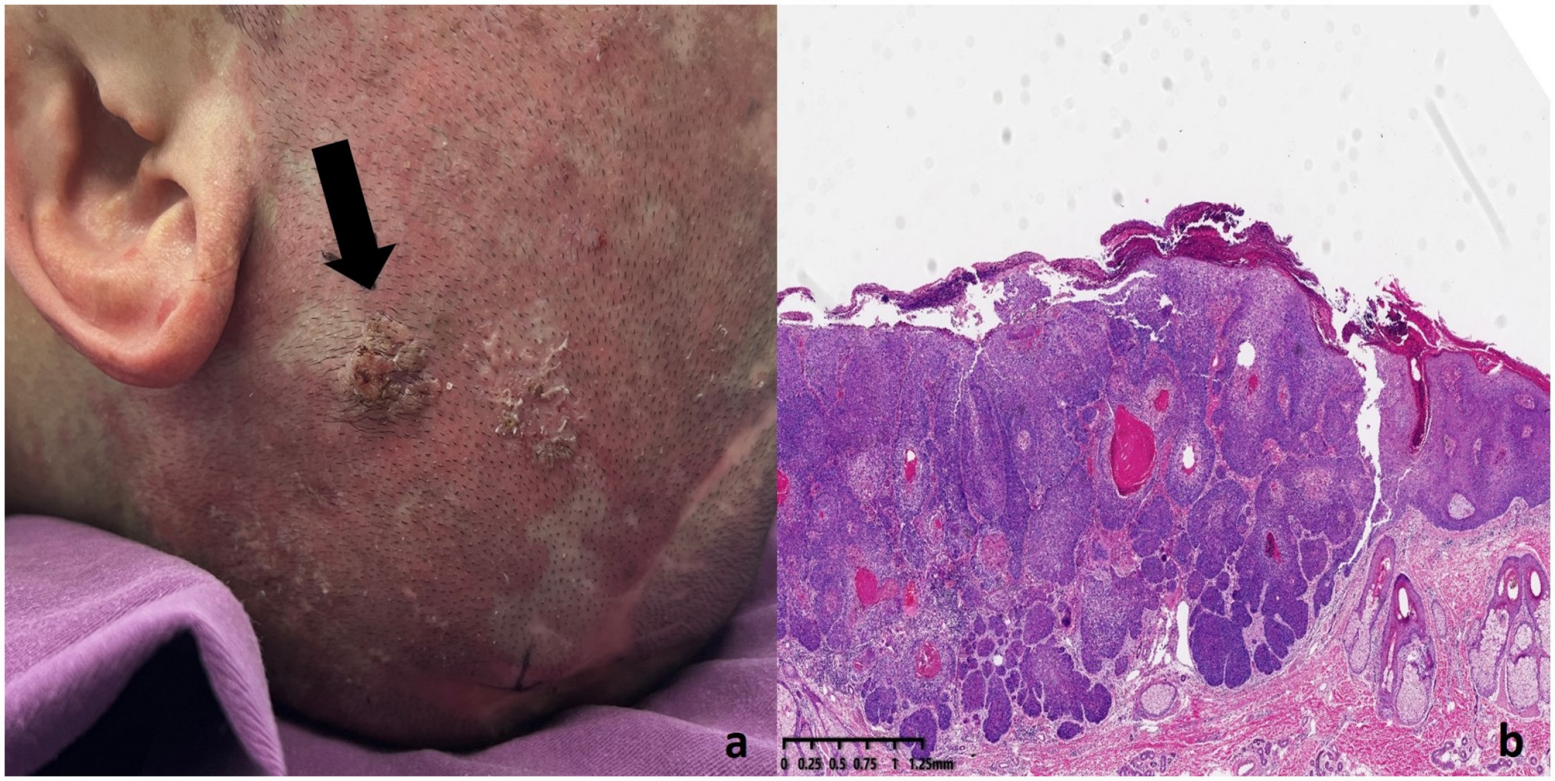
SCC lesion on the left temporal region of the patient **(a)** Histopathology of SCC lesions. **(b)** The tumor cells were well-differentiated, resembling normal squamous epithelium. The cytoplasm was abundant, and the nuclei showed mild atypia. Intercellular bridges and keratin pearls were observed, confirming the diagnosis of well-differentiated squamous cell carcinoma. The tumor had infiltrated the deep dermis with chronic inflammatory cells present in the surrounding tissue.

Multiple keratotic masses were observed on the left temporal region of the patient, presenting as nodular lesions with a firm texture and well-defined borders. The largest lesion measured 1.2 cm in diameter (Figure 3a). Histopathological examination (Figure 3b) revealed a well-differentiated squamous cell carcinoma. The tumor cells were arranged in nests with good differentiation, closely resembling normal squamous epithelium in morphology. The cytoplasm was abundant, and the nuclei showed mild atypia. Prominent intercellular bridges and characteristic keratin pearls were observed, supporting the diagnosis of well-differentiated squamous cell carcinoma. The tumor infiltrated into the deep dermis and was accompanied by a chronic inflammatory cell infiltration in the surrounding tissue. No definite vascular or perineural invasion was identified.

**Figure 4:**
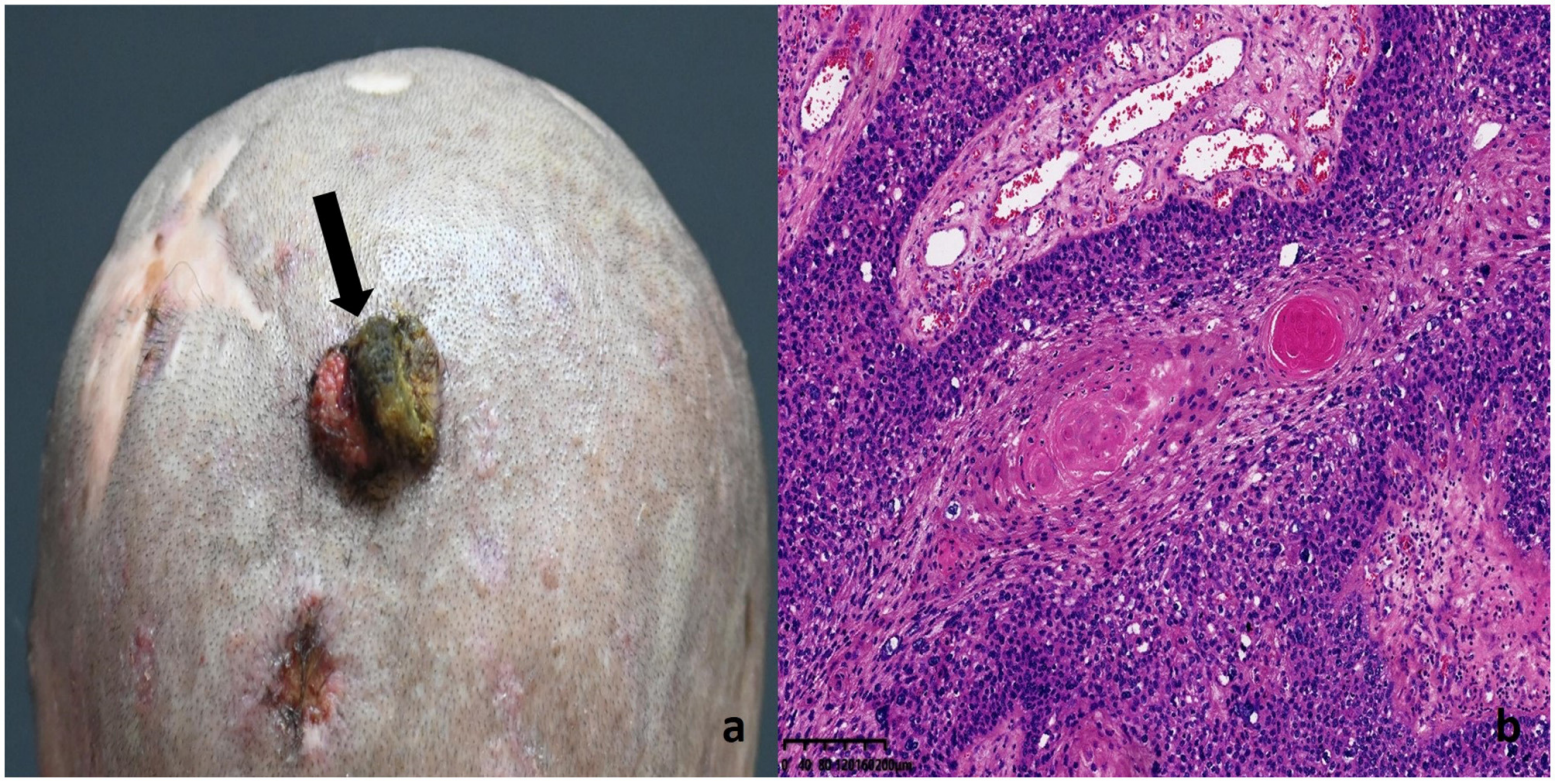
SCC lesion on the patient's occipital region: **(a)** Histopathology of SCC lesions **(b)** Tumor cells were arranged in nests and sheets, with invasive growth into the deep dermis. The cells were polygonal, with abundant eosinophilic cytoplasm, large atypical nuclei, prominent nucleoli, and frequent mitotic figures. Keratin pearls were observed at the center of the tumor nests, showing concentrically layered keratinized cells. The boundary between tumor parenchyma and stroma was well-defined, and intercellular bridges were clearly visible, indicating a relatively high degree of differentiation. The stroma showed varying degrees of lymphocytic infiltration, with areas of necrosis and inflammatory response.

A large, cauliflower-like exophytic mass was observed in the occipital region, measuring approximately 3.0 × 2.5 × 1.0 cm. The surface of the lesion showed ulceration and erosion, and it bled easily upon contact (Figure 4a). Histopathological examination revealed a moderately to well-differentiated squamous cell carcinoma (Figure 4b). Tumor cells were arranged in nests and sheets with invasive growth into the deep dermis. The cells were polygonal with abundant eosinophilic cytoplasm, large and atypical nuclei, prominent nucleoli, and frequent mitotic figures. Keratin pearls were observed in the center of tumor nests, showing concentrically layered keratinized cells. The boundary between tumor parenchyma and stroma was well-defined, and intercellular bridges were clearly visible, indicating a relatively high degree of differentiation. The stroma exhibited varying degrees of lymphocytic infiltration, with areas of necrosis and inflammatory response.

**Figure 5:**
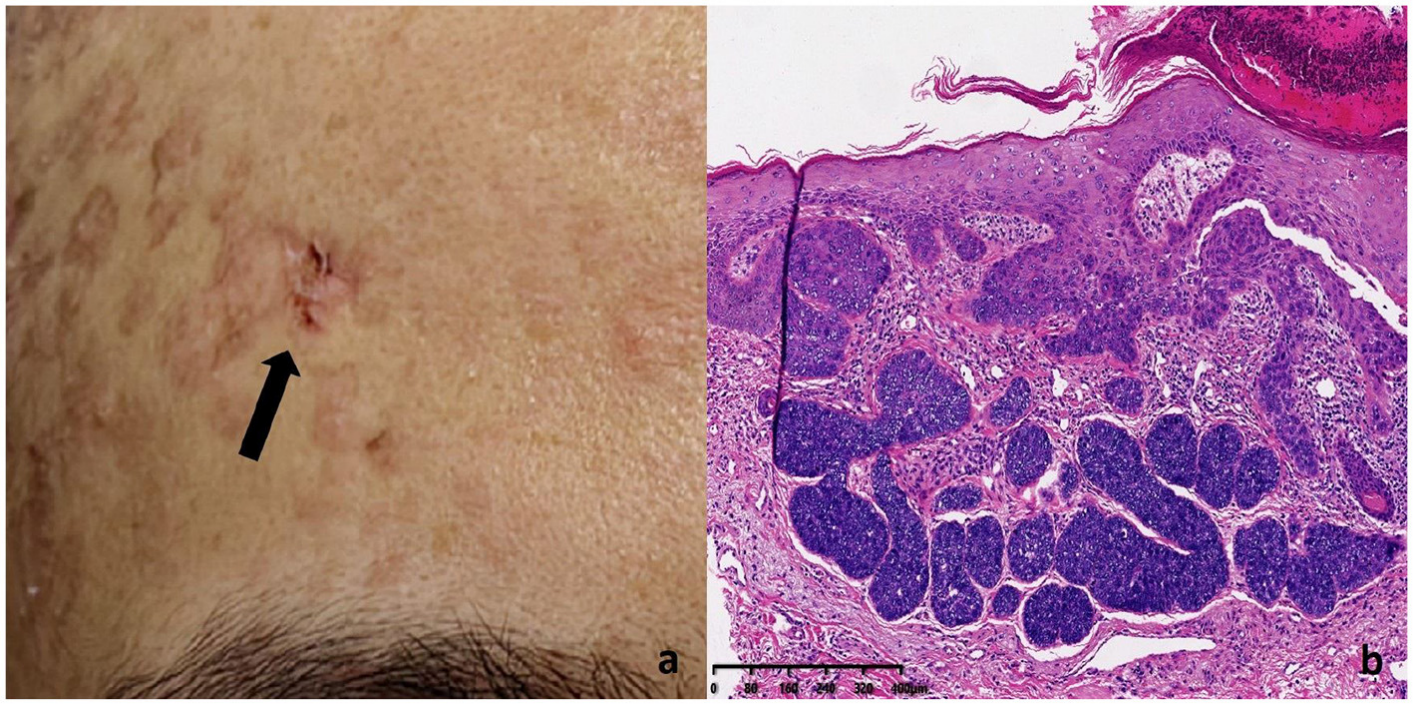
BCC lesion on the patient's right frontal region: **(a)** Histopathology of BCC lesions **(b)** Tumor cells exhibited basaloid features, characterized by hyperchromatic nuclei and scant cytoplasm, arranged in solid nests. Peripheral palisading of tumor cells was evident, and retraction artifacts were frequently observed, presenting as spaces that separate the tumor nests from the surrounding stroma.

In addition, a slightly elevated, brownish nodule approximately 0.5 cm in diameter was noted on the right frontal region, resembling a crusted wound without tenderness on palpation (Figure 5a). Histopathological analysis confirmed basal cell carcinoma (Figure 5b). Tumor cells exhibited basaloid features, with hyperchromatic nuclei and scant cytoplasm, arranged in solid nests. Palisading of the peripheral tumor cells was evident, and retraction artifacts, manifested as spaces separating the tumor nests from the surrounding stroma, were frequently observed.

**Figure 6:**
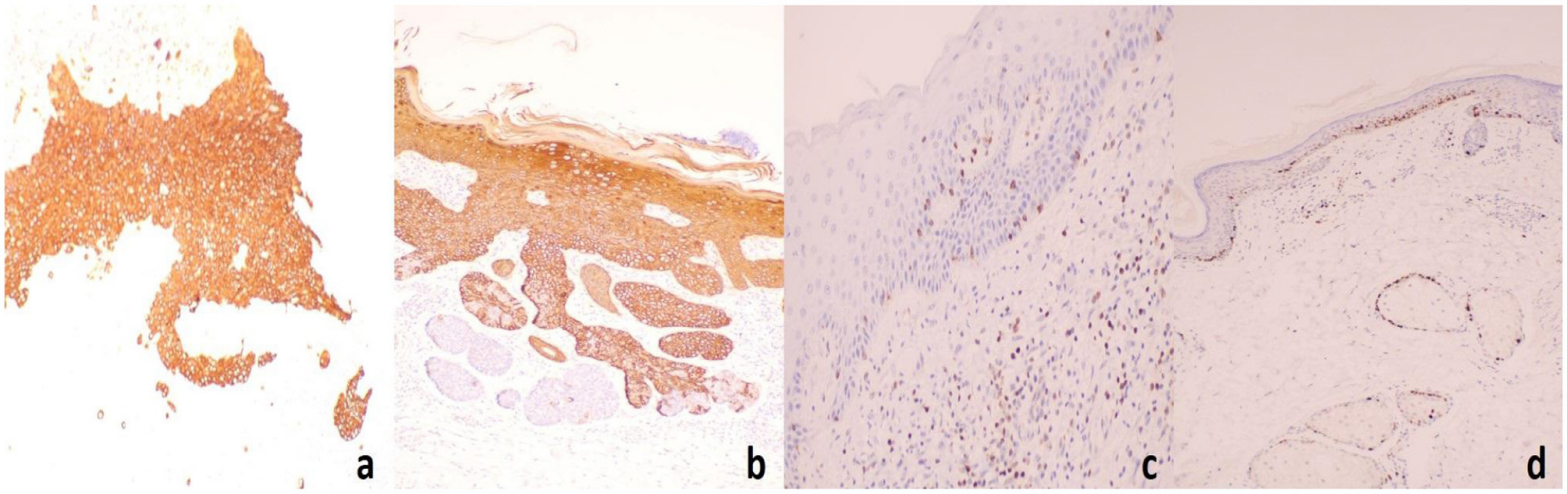
Immunohistochemistry results for the right frontal lobe: **(a)** CK5/6(+), **(b)** CK14(+), **(c)** BCL-2(+), **(d)** Ki-67(+) at 60%.

The complete blood count (CBC) and biochemical results were within normal limits. HPV genotyping in exfoliated cells from the frontal and temporal lobes revealed negative results for all 21 types of HPV. HIV antibodies: negative. Syphilis antibodies: negative. Immunohistochemistry results for the right frontal lobe: CK5/6 (+), CK14 (+), CD10 (−), CK20 (−), BCL-2 (+), Ki-67 (+) at 60% (Figure 6).

Transcriptome sequencing results: Mutations were detected in the TMC6/EVER1 and TMC8/EVER2 genes. The variant sites were annotated at the DNA level according to the HGVS standards as a heterozygous insertion variant (rs770886873) located at position 78,106,055 on chromosome 17 (reference genome version GRCh37). This mutation involved a G-to-GA change in the 3' untranslated region (3' UTR) of the TMC6 gene transcript, specifically a thymine repeat (c.*7092dupT). The mutation annotation indicates that the variant is downstream of the gene and may have a minimal or no effect on protein function. The sequencing depth at this site was 18 × , with a heterozygous genotype (0/1) supported by 7 sequencing reads for the reference allele and 9 reads for the mutant allele.

Similarly, at position 78,140,326 on chromosome 17 (reference genome version GRCh37), a heterozygous insertion variant (rs34749218) was detected, involving a C-to-CA insertion mutation. This mutation is located in the intronic region of the TMC8 gene, with the insertion variant annotated as c.1234-508_1234-507insA. The sequencing depth at this site was 54.64 × , with a heterozygous genotype (0/1) supported by 15 sequencing reads for the reference allele and 5 reads for the mutant allele.

Based on the patient's pathological findings, clinical history, and laboratory tests, the diagnosis is Epidermodysplasia Verruciformis with concomitant squamous cell carcinoma and basal cell carcinoma.

**Figure 7:**
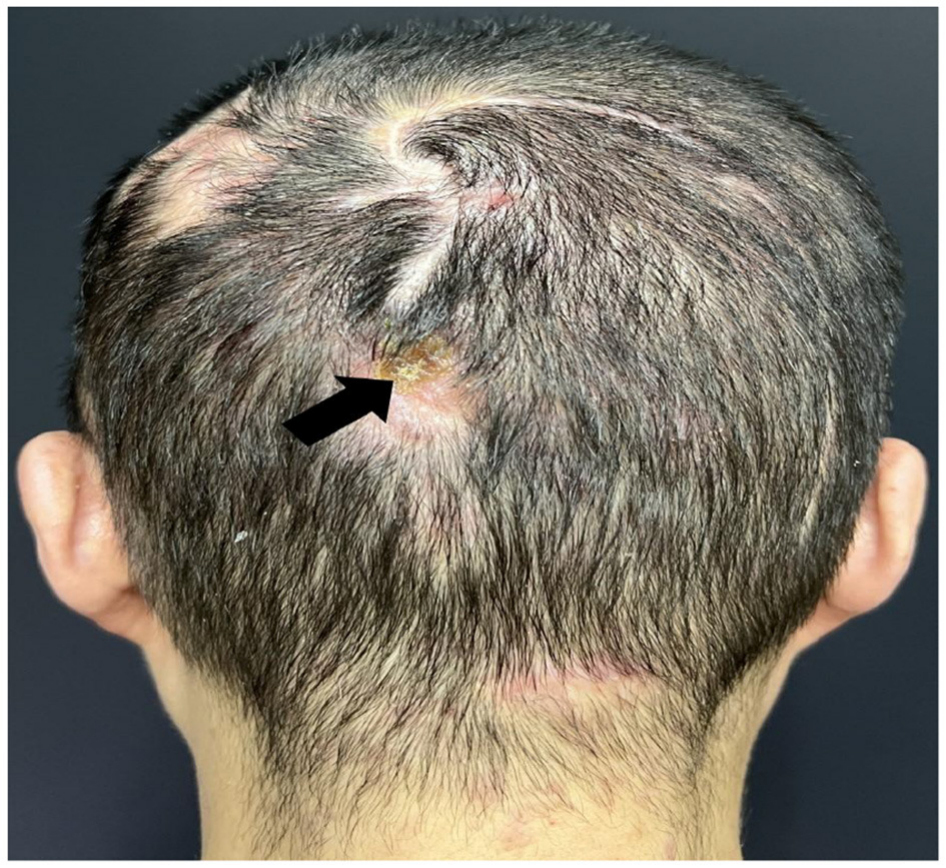
Postoperative condition one year after surgery: a seborrheic keratosis-like lesion was observed on the occipital region (arrows), with good healing of the surgical incision.

For the five prominent proliferative lesions located on the patient's head and face, surgical excision was performed. Intraoperative frozen section analysis confirmed that all resection margins were free of cancerous involvement. Postoperative histopathological analysis also revealed clear surgical margins with no evidence of perineural or vascular invasion. The patient recovered well without the need for adjuvant therapy or medication. At the one-year follow-up, no significant progression or tumor recurrence was observed, except for a seborrheic keratosis-like lesion on the occipital region (Figure 7). Lesions presenting with rapid enlargement and seborrheic keratosis-like features should be carefully evaluated due to their potential for malignant transformation, and early surgical excision is recommended in such cases. Comprehensive dermatological and surgical evaluations revealed no signs of regional lymphadenopathy or distant metastases. We advise regular outpatient follow-up to closely monitor the patient's condition and ensure timely intervention if necessary.

## 3 Discussion

Epidermodysplasia verruciformis (EV) is characterized primarily by wart-like lesions on the skin, typically occurring in sun-exposed areas. The condition often has a genetic basis, with inherited susceptibility increasing the risk of developing multiple wart-like lesions and precancerous changes. Over time, these lesions may progress to cutaneous squamous cell carcinoma (SCC) (6).

Current research indicates that EV is primarily caused by autosomal recessive mutations in the EVER1 (TMC6) or EVER2 (TMC8) genes. These genes play a key role in the immune response to human papillomavirus (HPV), particularly to oncogenic cutaneous HPV types such as HPV 5 and HPV 8. Mutations in EVER1 (TMC6) or EVER2 (TMC8) impair the host's ability to mount an effective immune defense against HPV infection, making affected individuals highly susceptible to persistent HPV colonization (7).

Mutations in the EVER1 (TMC6) and EVER2 (TMC8) genes are clinically associated with autosomal recessive epidermodysplasia verruciformis (AR-EV). In most cases, the inheritance pattern involves homozygous mutations, meaning that affected individuals typically carry two mutated alleles. Heterozygous carriers, in contrast, are usually asymptomatic but can pass the mutated gene to their offspring. Although EV is predominantly inherited in an autosomal recessive manner, some cases have been reported with compound heterozygous mutations (7–9).

Unlike typical cases of EV with autosomal recessive inheritance, the patient in this case developed symptoms at a young age without a family history of related genetic disorders. In this case, transcriptome sequencing identified two heterozygous variants in genes classically associated with epidermodysplasia verruciformis: TMC6 (EVER1) and TMC8 (EVER2). The TMC6 variant (rs770886873; c.*7092dupT) is a single-nucleotide insertion located in the 3' untranslated region (3'UTR) of the gene, while the TMC8 variant (rs34749218; c.1234-508_1234-507insA) lies within an intronic region. Although these variants do not affect the coding sequence directly, non-coding mutations in the 3'UTR or introns may disrupt post-transcriptional regulatory mechanisms, such as mRNA stability, splicing, or microRNA binding. As the EVER1 and EVER2 proteins are known to form a functional complex involved in zinc homeostasis and keratinocyte-intrinsic immunity to β-HPV, even subtle disruption of their expression or regulation may compromise host defense and contribute to EV pathogenesis (10). Importantly, no mutations were detected in other known EV-associated or immunodeficiency-related genes, such as RHOH, IL7, CORO1A, STK4, DOCK8, or CARMIL2, supporting the notion that the identified TMC6 and TMC8 variants may represent the primary genetic contributors in this case (11–13).

In this context, we propose that the two gene products may function within the same metabolic pathway, and that the effects of their respective mutations may be additive. If the biological processes involving the proteins encoded by these genes are highly sensitive to functional impairment, even a partial loss of function, without complete homozygous inactivation of both genes, may be sufficient to disrupt normal cellular function and lead to disease manifestation.

In this case, exfoliated cells from the patient's forearm were collected for polymerase chain reaction (PCR)-based HPV DNA genotyping, targeting common mucosal types including HPV 6, 11, 16, 18, 31, 33, 35, 39, 42, 43, 45, 51–53, 56, 58, 59, 66, 68, 73, and 81. All tested types were negative, preliminarily ruling out the possibility of high-risk cervical cancer-associated HPV types being the causative agents. Whether this finding aligns with reports of EV cases associated with high-risk HPV subtypes in malignant lesions, as described in both domestic and international literature, remains to be further investigated. EV-associated squamous cell carcinoma (SCC) is most commonly linked to HPV types 3, 5, 6, 8, 14, 20, 46, and 47 (14). Among these, HPV types 5, 8, 10, and 47 have been identified in over 90% of EV-related malignant skin lesions, whereas HPV types 14, 20, 21, and 25 are typically associated with benign lesions only (15). Due to limitations in testing resources, the current PCR panel did not include HPV types 3, 5, 8, or 10. Therefore, whether the patient's lesions are directly attributable to infection with these specific EV-associated HPV types requires further molecular investigation.

EV requires differential diagnosis from various skin conditions that present with similar features, particularly those characterized by multiple flat papules or wart-like plaques. Common differential diagnoses include common warts, flat warts, Darier's disease, lichen planus, seborrheic keratosis, psoriasis, and early-stage squamous cell carcinoma (SCC) or basal cell carcinoma (BCC). Among these, flat warts are similar to EV in terms of morphology and distribution, necessitating HPV typing for differentiation. While Darier's disease and lichen planus may also present with keratotic papules, they exhibit characteristic nail changes or mucosal involvement, respectively. Seborrheic keratosis and psoriasis differ in clinical presentation, age of onset, and lesion characteristics. Additionally, early-stage skin cancers such as Bowen's disease may present with similar lesions, requiring skin biopsy for confirmation. Auxiliary examinations, including HPV typing, histopathological analysis, and screening for mutations in EVER1 (TMC6) or EVER2 (TMC8) genes, are essential for confirming the diagnosis of EV and excluding other conditions (16, 17).

This case report describes a 38-year-old male patient with a long-standing history of EV, which progressed to multiple malignancies, including SCC and BCC. This condition is extremely rare, especially in a patient with multiple skin cancers arising from EV, highlighting the need for increased awareness of this diagnosis and consideration in patients presenting with similar lesions. The report provides valuable insight for clinicians in diagnosing and managing similar cases, underscoring the importance of proactive monitoring in EV patients to detect and address potential malignant transformations in a timely manner.

## Data Availability

The raw data supporting the conclusions of this article will be made available by the authors, without undue reservation.
