# Correction: Case Report: Epidermodysplasia verruciformis with multiple squamous and basal cell carcinomas

**DOI:** 10.3389/fmed.2026.1802415

**Published:** 2026-02-17

**Authors:** Zijing Zhang, Chuanbo Liu, Jinsheng Li

**Affiliations:** 1 The Fourth School of Clinical Medicine, Zhejiang Chinese Medical University, Hangzhou First People's Hospital, Hangzhou, China; 2 Department of Plastic and Cosmetic Surgery, Affiliated Hangzhou First People's Hospital, School of Medicine, Westlake University, Hangzhou, Zhejiang, China

**Keywords:** epidermodysplasia verruciformis, squamous cell carcinoma, basal cell carcinoma, histopathological examination, malignancy

Affiliation 1 “The Fourth School of Clinical Medicine, Zhejiang Chinese Medical University, Hangzhou First People's Hospital, Hangzhou, China” was erroneously given as “The Fourth Clinical Medical College, Zhejiang Chinese Medicine University, Hangzhou, China”.

Affiliation 2 “Department of Plastic and Cosmetic Surgery, Affiliated Hangzhou First People's Hospital, School of Medicine, Westlake University, Hangzhou, Zhejiang, China” was erroneously given as “Department of Plastic and Cosmetic Surgery, Affiliated Hangzhou First People's Hospital, Hangzhou, Zhejiang, China”.

The funder “Hangzhou Biomedical and Health Industry Development Support Science and Technology Program, 2024WJC100 to Jinsheng Li” was erroneously omitted. The correct Funding statement appears below:

“The author(s) declared that financial support was received for this work and/or its publication. This work was supported by the Hangzhou Biomedical and Health Industry Development Support Science and Technology Program, number 2024WJC100 to JL.”

The original version of this article has been updated.

