# Supplementary figures and images for: Case Report: Epidermodysplasia verruciformis with multiple squamous and basal cell carcinomas
Source: Front Med (Lausanne). 2025 Jun 4;12:1565977. doi: 10.3389/fmed.2025.1565977 (PMC12173910; doi:10.3389/fmed.2025.1565977)

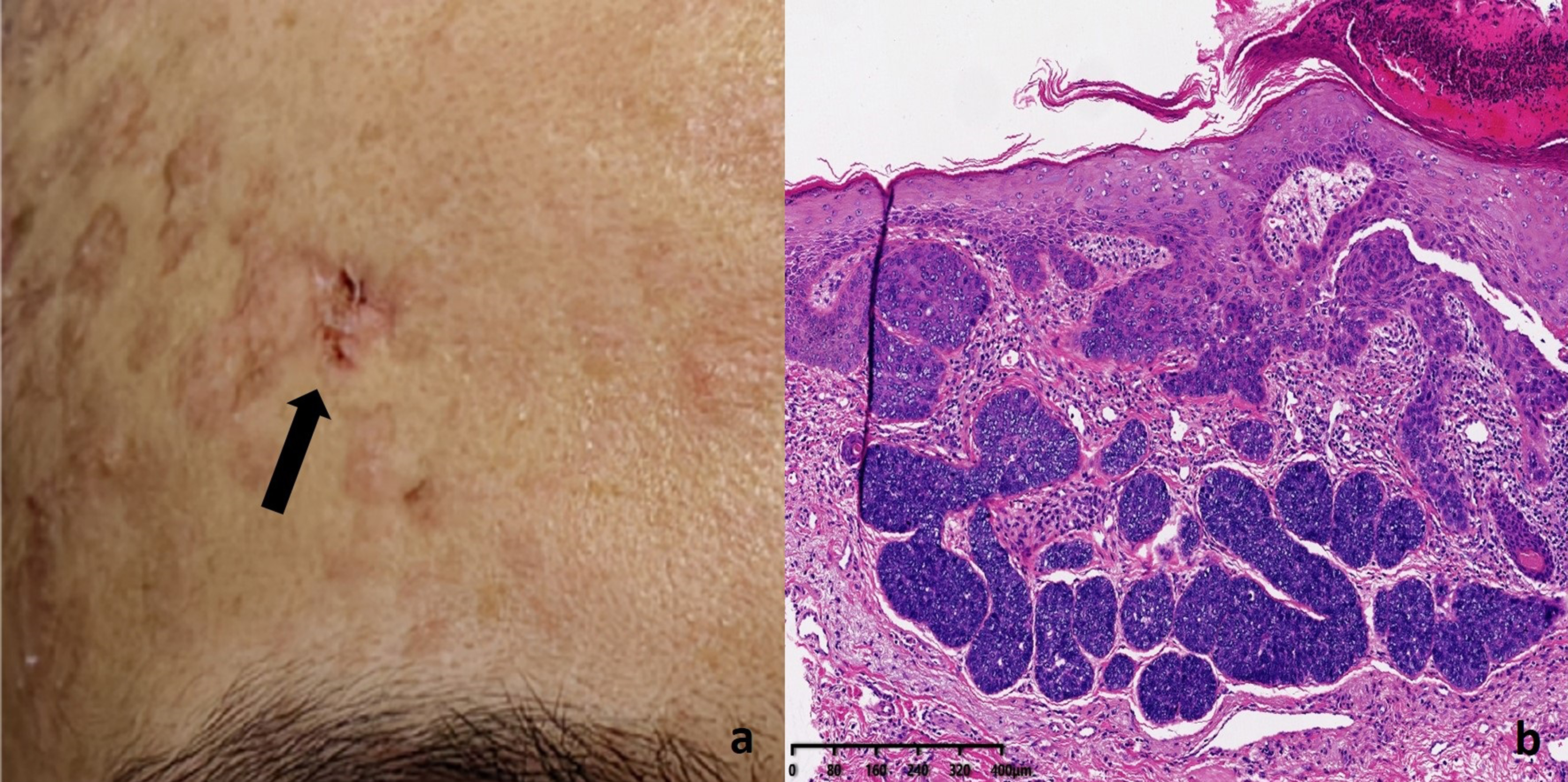

Supplement: Supplementary file 1 [file Image_1.jpeg]

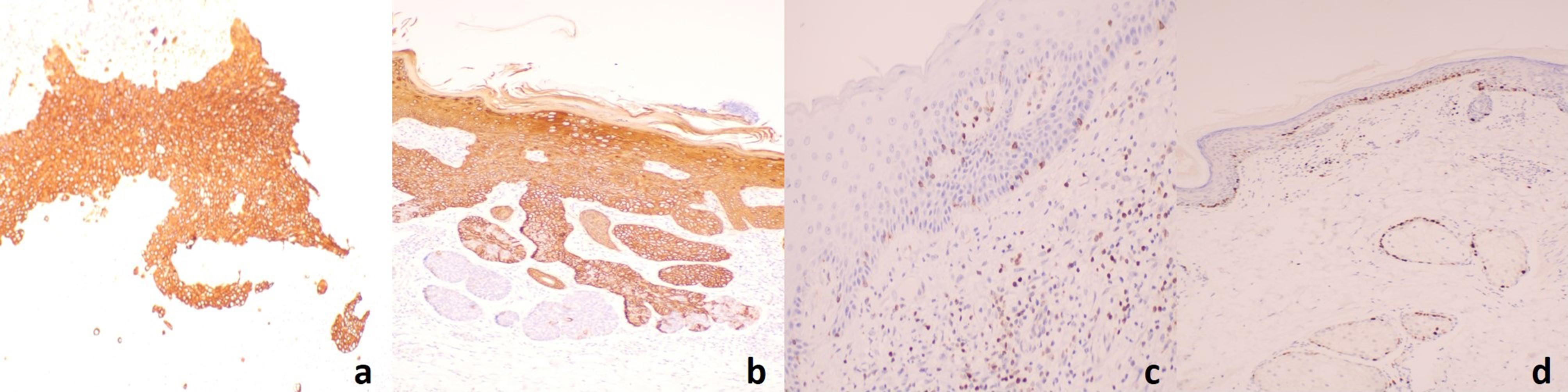

Supplement: Supplementary file 2 [file Image_2.jpeg]

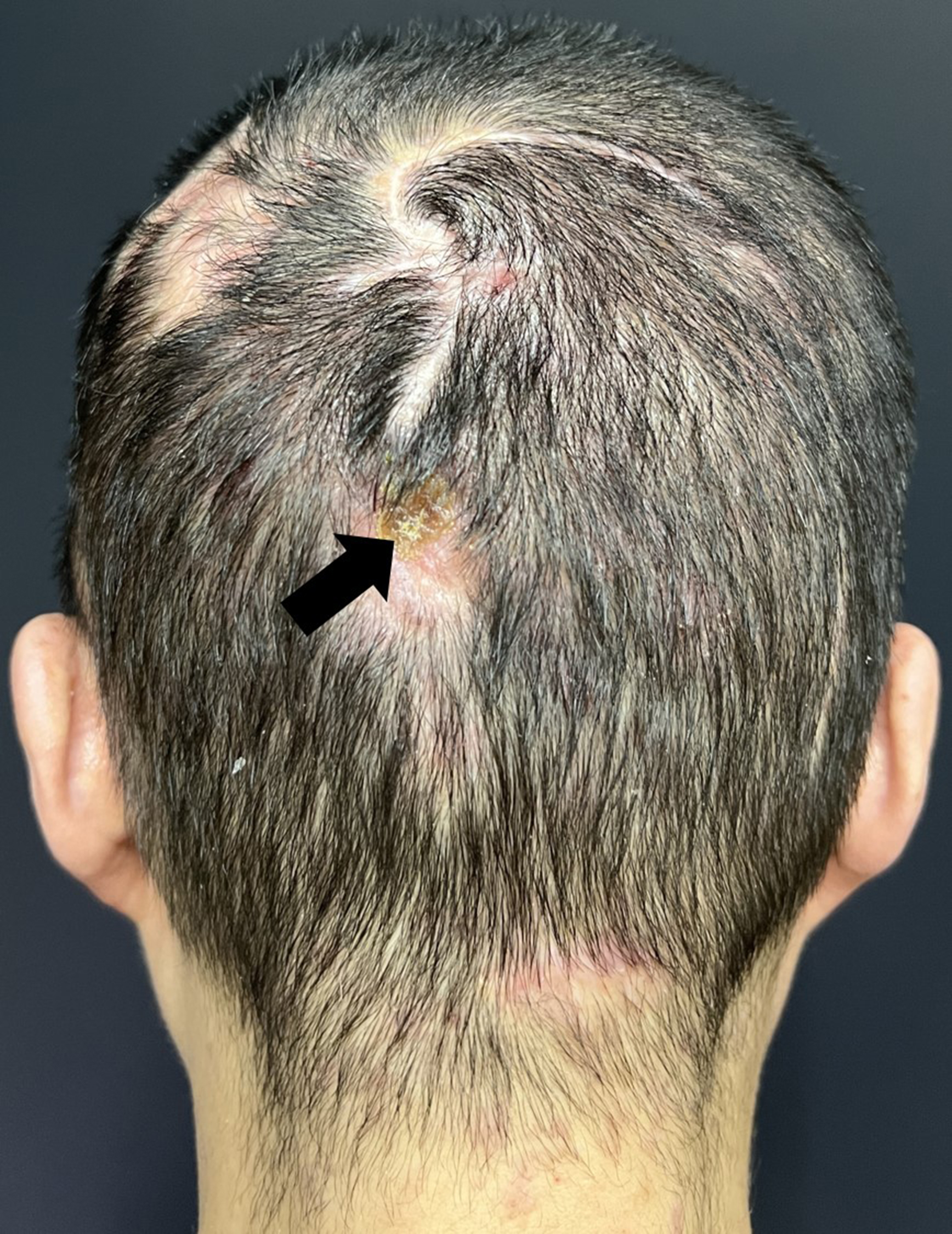

Supplement: Supplementary file 3 [file Image_3.jpeg]
